# Asthma increases short-time sickness absence and presenteeism in young adults

**DOI:** 10.1016/j.jacig.2025.100518

**Published:** 2025-06-24

**Authors:** Inger Kull, Maria Ödling, Ida Mogensen, Anne-Sophie Merritt, Theo Bodin, Gun Johansson, Sandra Ekström

**Affiliations:** a Department of Clinical Science and Education, Södersjukhuset, Karolinska Institutet, Stockholm, Sweden; b Sachs' Children and Youth Hospital, Södersjukhuset, Karolinska Institutet, Stockholm, Sweden; c Department of Women’s and Children’s Health, Karolinska Institutet, Stockholm, Sweden; e Institute of Environmental Medicine, Karolinska Institutet, Stockholm, Sweden; d Center for Occupational and Environmental Medicine, Region Stockholm, Stockholm, Sweden

**Keywords:** Asthma, presenteeism, allergic rhinitis, sickness absence, young adults

## Abstract

**Background:**

There is limited knowledge on how asthma affects short-term sickness absence and presenteeism among young adults.

**Objective:**

Our aim was to investigate associations between asthma and short-term sickness absence and presenteeism among young adults. A secondary aim was to investigate the influence of comorbidity with rhinitis and skill levels in occupations for these associations.

**Methods:**

The study included 2029 participants from the population-based Barn/Children Allergy Milieu Stockholm Epidemiology (BAMSE) cohort who answered questions on allergic disease at age 24 years and questions on work-related outcomes in a follow-up at age 28 years. Short-term sickness absence was defined as sickness absence lasting fewer than 14 days. Presenteeism was defined as working despite being unwell. Analyses were performed with multinominal logistic regression (sickness absence) and logistic regression (presenteeism) adjusted for sex, education, employment status, smoking status, and body mass index.

**Results:**

The participants with current asthma reported more short-term sickness absence and presenteeism, respectively, than did those without current asthma (eg, 37.2% vs 30.5% reported 2-4 episodes and 8.6% vs 5.7% reported ≥5 episodes in the preceding 12 months [*P* = .01]). In multivariate analyses, current asthma was associated with frequent sickness absence (relative risk ratio = 1.37 [95% CI = 1.02-1.84 for 2-4 episodes] and relative risk ratio = 1.74 [95% CI = 1.03-2.94 for ≥5 episodes]), as well as higher presenteeism (odds ratio = 1.40 [95% CI = 1.06-1.85]). For the most frequent sickness absence category (≥5 episodes), there was an association for asthma with, but not without, allergic rhinitis and a tendency for a greater association in occupations with low- versus high-skill levels.

**Conclusion:**

Asthma is associated with frequent short-term sickness absence and presenteeism among young adults. Optimal treatment and management of asthma may reduce its negative consequences on working life.

## Introduction

The prevalence of asthma is around 10% in Western countries, and a large proportion of individuals with asthma also experience allergic rhinitis.^1^ Previous research has shown that a large proportion of adolescents and young adults with asthma experience inadequate disease control, limited health care contact, and few dispensed medications.^2^^,^^3^ Additionally, previous studies, including our Barn/Children Allergy Milieu Stockholm Epidemiology (BAMSE) cohort, highlight deficiencies in the transition process from pediatric to adult health care, with even further reduction in health care consumption and medication use.^4^, ^5^, ^6^ Ultimately, this may lead to considerable health care costs, reduced quality of life, and loss of work productivity among young individuals with asthma.

Asthma has been shown to negatively affect several aspects of working life, including sickness absence, disability pension, and unemployment.^7^, ^8^, ^9^ In a recent study from our group, asthma was found to be associated with a higher rate of longer periods of sickness absence (>14 days) among young adults, especially in those with uncontrolled asthma, persistent asthma, and asthma with rhinitis.^10^

A drawback with most previous studies^7^^,^^8^^,^^10^ is that information on sickness absence was register based, as a result of which only longer periods of sickness absence (>14 days) are captured. We assume that asthma mainly affects short-term sickness absence due to exacerbations during respiratory infections and shorter periods of high allergen exposure.^11^^,^^12^ These exacerbations may also lead to increased presenteeism (ie, working when unwell). Presenteeism can have negative consequences on work performance and health,^13^ and it has been shown to be even more related to asthma control than absenteeism.^14^

However, few previous studies have investigated the association between asthma and short-time sickness absence and presenteeism, especially in young adults. The aim of the present study was therefore to investigate associations between asthma and frequent short-term sickness absence as well as presenteeism. A secondary aim was to investigate whether comorbidity with allergic rhinitis and skill levels in occupations may influence these potential associations.

For detailed information on methods, please see the Online Repository (available at www.jaci-global.org).

## Results and discussion

In total, 2029 participants (59.2% female) had complete information on asthma, sickness absence, presenteeism, and covariates (sex, education, employment status, smoking, and body mass index) and were included in the analyses. Most participants (62.1%) had a university education (≥3 years), and 78.2% reported working as their main occupation. The majority of participants (59.3%) lived with a partner; 31.6% lived alone. Females were more likely than males to have a university education (≥3 years); there was no sex difference in terms of employment status. In total, 63.8% of the study population reported at least 1 episode with sickness absence and 60.3% reported at least 1 episode with presenteeism in the preceding 12 months. Females were more likely than males to report any sickness absence (67.7% vs 58.2% [*P* < .001]) and any presenteeism (64.8% vs 53.7% [*P* < .001]). For additional descriptions of the study population, see Table I.

**Table I:** Description of the study population

Characteristic	Females (n = 1202)	Males (n = 827)	Total (N = 2029)	*P* value
Age (y), mean (SD)	28.4 (0.8)	28.4 (0.8)	28.4 (0.8)	1.00
Education level, no. (%)				
Elementary school/upper secondary school	214 (17.8)	244 (29.5)	458 (22.6)	<.001
Folk high school/university <3 y	187 (15.6)	124 (15.0)	311 (15.3)	
University ≥ 3 y	801 (66.6)	459 (55.5)	1,260 (62.1)	
Employment status, no. (%)				
Studying	172 (14.3)	125 (15.1)	297 (14.6)	.40
Working	936 (77.9)	650 (78.6)	1,586 (78.2)	
Other	94 (7.8)	52 (6.3)	146 (7.2)	
Living conditions (n = 2027), no. (%)∗				
Live alone	339 (28.3)	302 (36.5)	641 (31.6)	<.001
Live together with parents or siblings	70 (5.8)	86 (10.4)	156 (7.7)	<.001
Live together with partner	773 (64.4)	428 (51.8)	1,201 (59.3)	<.001
Live together with other adults	37 (3.1)	31 (3.8)	68 (3.4)	.41
Live together with children	124 (10.3)	55 (6.7)	179 (8.8)	.004
Sickness absence <14 d in past 12 mo, no. (%)				
Never	388 (32.3)	346 (41.8)	734 (36.2)	<.001
Once	311 (25.9)	227 (27.5)	538 (26.5)	
2-4 times	418 (34.8)	217 (26.2)	635 (31.3)	
5-9 times	68 (5.7)	31 (3.8)	99 (4.9)	
≥10 times	17 (1.4)	6 (0.7)	23 (1.1)	
Presenteeism in past 12 mo, no. (%)				
Never	423 (35.2)	383 (46.3)	806 (39.7)	<.001
Once	305 (25.4)	184 (22.3)	489 (24.1)	
A few times	398 (33.1)	230 (27.8)	628 (31.0)	
Several times	76 (6.3)	30 (3.6)	106 (5.2)	
Influence of the pandemic on working situation (n = 2026)∗				
Changed occupation	170 (14.2)	111 (13.4)	281 (13.9)	.64
Was or is unemployed	58 (4.8)	50 (6.1)	108 (5.3)	.23
Status based on body mass index				
Underweight (<18.5 kg/m^2^)	40 (3.3)	15 (1.8)	55 (2.7)	<.001
Normal weight (18.5-24.9 kg/m^2^)	822 (68.4)	474 (57.3)	1,296 (63.9)	
Overweight (25-29.9 kg/m^2^)	229 (19.1)	270 (32.7)	499 (24.6)	
Obese (≥ 30 kg/m^2^)	111 (9.2)	68 (8.2)	179 (8.8)	
Smoking				
No	1,096 (91.2)	759 (91.8)	1,855 (91.4)	.66
Occasionally	77 (6.4)	53 (6.4)	130 (6.4)	
Daily	29 (2.4)	15 (1.8)	44 (2.2)	
Asthma at age 24 y, no. (%)	152 (12.7)	82 (9.9)	234 (11.5)	.06

∗ Several alternatives are possible.

**Fig 1:**
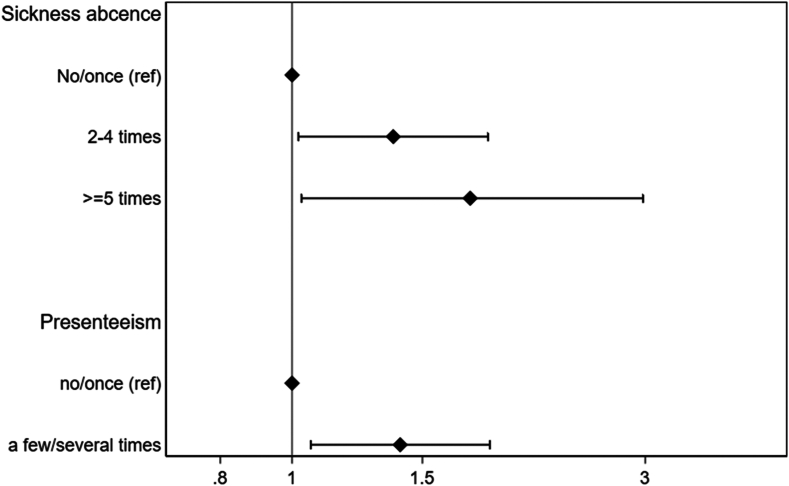
Associations between current asthma at age 24 years and number of times with sickness absence (RRR) as well as with presenteeism (OR) in the past 12 months at age 28 years (N = 2029). Analyses were performed with multinomial logistic regression (sickness absence) and logistic regression (presenteeism) adjusted for sex, education, occupation, smoking, and body mass index.

Frequent short-term sickness absence at age 28 years was more common among individuals with current asthma than among those without current asthma at age 24 years (37.2% vs 30.5% had 2-4 episodes and 8.6% vs 5.7% had ≥5 episodes in the preceding 12 months [*P* = .01]). Similarly, individuals with current asthma at age 24 years had more presenteeism at age 28 years than did those without current asthma (43.6% vs 35.2% [*P* = .01] with a few to several times in the preceding 12 months). After adjustment for confounders (Fig 1), current asthma was associated with frequent sickness absence (relative risk ratio [RRR] = 1.37 [95% CI = 1.02-1.84 for 2-4 episodes] and RRR = 1.74 [95% CI = 1.03-2.94 for ≥5 episodes]), as well as higher presenteeism (odds ratio [OR] = 1.40 [95% CI = 1.06-1.85]) than among those participants without current asthma. There was no significant difference in the results with stratification by sex.

Analyzing current asthma in combination with allergic rhinitis (n = 2011) showed that asthma with allergic rhinitis was associated with 5 or more episodes of sickness absence in the preceding 12 months (RRR = 2.42 [95% CI = 1.33-4.42]), whereas asthma without allergic rhinitis was associated with 2 to 4 episodes of sickness absence in the preceding 12 months (Table II). Asthma with allergic rhinitis was further associated with increased presenteeism (OR = 1.66 [95% CI = 1.17-2.37] for a few or several times in the preceding 12 months). Similar results were observed for allergic rhinitis without asthma (OR = 1.42 [95% CI = 1.14-1.78]), whereas no significant association was observed for asthma without allergic rhinitis (Table III).

**Table II:** Associations between current asthma in combination with allergic rhinitis at age 24 years and short-time sickness absence at age 28 years (n = 2011)

Asthma phenotype	Sickness absence <14 d, No. of times in past 12 mo	
	2-4 times	≥5 times
	RRR (95% CI)	RRR (95% CI)
No asthma, no allergic rhinitis (n = 1319)	Reference value	Reference value
No asthma, allergic rhinitis (n = 460)	1.08 (0.86-1.37)	1.34 (0.85-2.10)
Asthma, no allergic rhinitis (n = 89)	**1.85 (1.18-2.91)**	1.07 (0.37-3.12)
Asthma and allergic rhinitis (n = 143)	1.15 (0.78-1.69)	**2.42 (1.33-4.42)**

Asthma combined with rhinitis was analyzed by creating a categoric variable (no asthma and no rhinitis [reference value]), no asthma but rhinitis, asthma but no rhinitis, and asthma with rhinitis. Analyses were performed with multinomial logistic regression. RRRs were adjusted for sex, education, occupation, smoking, and body mass index. Boldface indicates statistical significance.

**Table III:** Associations between current asthma in combination with allergic rhinitis at age 24 years and presenteeism at age 28 years (n = 2011)

Asthma phenotype	Presenteeism, a few to several times in the past 12 m
	OR (95% CI)
No asthma, no allergic rhinitis (n = 1319)	Reference value
No asthma, allergic rhinitis (n = 460)	**1.42 (1.14-1.78)**
Asthma, no allergic rhinitis (n = 89)	1.43 (0.91-2.22)
Asthma and allergic rhinitis (n = 143)	**1.66 (1.17-2.37)**

Asthma combined with rhinitis was analyzed by creating a categoric variable (no asthma and no rhinitis [reference value]), no asthma but rhinitis, asthma but no rhinitis, and asthma with rhinitis. Analyses were performed with logistic regression. ORs were adjusted for sex, education, occupation, smoking, and body mass index. Boldface indicates statistical significance.

Stratification of the results for occupation (n = 1754) showed a tendency toward an association between current asthma and sickness absence in occupations with low skill levels (RRR = 1.48 [95% CI = 0.96-2.28]) but not in those with high skill levels (RRR = 1.02 [95% CI = 0.65-1.62]) for 2-4 episodes of sickness absence in the preceding 12 months) (Table IV). No difference in the association between current asthma and presenteeism was observed in relation to occupational skill level (Table V).

**Table IV:** Associations between current asthma at age 24 years (2016-2019) and short-time sickness absence at age 28 years (2023), stratified by occupational skill level (in 2021) (n = 1754)

Asthma status	Sickness absence <14 days, No. of times in the past 12 mo			
	2-4 times		≥5 times	
	High skill level (SSYK 1-3)	Low skill level (SSYK 4-9)	High skill level (SSYK 1-3)	Low skill level (SSYK 4-9)
	RRR (95% CI)	RRR (95% CI)	RRR (95% CI)	RRR (95% CI)
No asthma	Reference value	Reference value	Reference value	Reference value
Asthma	1.02 (0.65-1.62)	1.48 (0.96-2.28)	1.48 (0.65-3.36)	1.88 (0.91-3.85)

SSYK 1 to 3 refers to managerial occupations and occupations with university qualifications (skill level 3-4; n = 837), SSYK 4 to 9 refers to occupations in administration, service, care, and sales work, agriculture, gardening, forestry, fishing, construction, manufacturing, transport, and occupations requiring shorter training (primary school level) (skill level 1-2; n = 917). Analyses were performed with multinomial logistic regression. RRRs were adjusted for sex, smoking, and body mass index (continuous).

*SSYK*, Swedish Standard Classification of Occupation.

**Table V:** Associations between current asthma at age 24 years (2016-2019) and presenteeism at age 28 years (2023), stratified by occupational skill level (in 2021) (n = 1754)

Asthma status	Presenteeism, a few to several times in the past 12 m	
	High skill level (SSYK 1–3)	Low skill level (SSYK 4–9)
	OR (95% CI)	OR (95% CI)
No asthma	Reference value	Reference value
Asthma	1.35 (0.88-2.06)	1.23 (0.81-1.85)

SSYK 1 to 3 refers to managerial occupations and occupations with university qualifications (skill level 3-4; n = 837), SSYK 4 to 9 refers to occupations in administration, service, care, and sales work, agriculture, gardening, forestry, fishing, construction, manufacturing, transport, and occupations requiring shorter training (primary school level) (skill level 1-2; n = 917). Analyses were performed with logistic regression. ORs were adjusted for sex, smoking, and body mass index (continuous).

*SSYK*, Swedish Standard Classification of Occupation.

In summary, the results from this population-based cohort showed that current asthma was associated with frequent short-term sickness absence and increased presenteeism. For the most frequent sickness absence category (≥5 episodes), there was an association with allergic rhinitis but not with nonallergic rhinitis. Moreover, there was a tendency for a more pronounced association with short-term sickness absence in occupations with a low versus high skill level.

The results of the present study are in line with those of previous studies, including those of our recently published study in which we found that asthma—especially persistent asthma, uncontrolled asthma, and asthma in combination with rhinitis—was associated with higher sickness absence (>14 days) and productivity losses.^10^ Moreover, a Finnish study of public sector employees showed associations between asthma as well as rhinitis and sickness absence, with the strongest association for both conditions combined.^9^ The results are also in line with those of a study of asthma cases in Australia, New Zealand, and Singapore, which showed that severe asthma was associated with increased presenteeism when compared with nonsevere asthma, although the study lacked a control group without asthma.^15^ The present study adds to the growing body of evidence demonstrating the negative impact of asthma on working life and suggests that individuals in low-skill occupations may be particularly affected.

Altogether, these results suggest that a considerable proportion of young adults with asthma do not fulfill the goal of asthma treatment, which involves good disease control and ability to live a life without restrictions due to their disease. This highlights the need for an effective transition process for adolescents with asthma to adult health care according to the European Academy of Asthma and Clinical Immunology guidelines^16^ in order to provide them with the skills required to self-manage their disease and avoid periods of exacerbation.^17^

The main strength of the study is its population-based prospective design, with asthma and rhinitis being assessed before the outcomes. The study’s limitations include the relatively few cases, thus limiting statistical power to investigate the role of asthma control and severity. Additionally, we used information on occupation from 2021 because no later data on the cohort were available and we lacked information on the causes of sickness absence and presenteeism. Moreover, as asthma and symptoms of allergic rhinitis were based on self-reports rather than on objective assessments, some misclassification cannot be ruled out. Finally, the generalizability of the findings to other countries may be limited owing to differences in factors such as health care and reimbursement systems, although patterns of sickness absence in Sweden have been shown to be similar to those in other Western countries.^18^

In conclusion, our results suggest that asthma is associated with frequent short-term sickness absence and presenteeism among young adults. As young adults with asthma have few health consultations and dispensed asthma medications,^19^ optimal treatment and management of asthma may be important to improving asthma control and thereby reducing asthma’s negative consequences on working life.

**Clinical implications**

Having asthma was associated with increased short-term sickness absence and presenteeism. To reduce the negative impact of asthma on working life, optimal treatment and effective management of the disease are needed.

## Disclosure statement

Supported by the Swedish Research Council; the Swedish Research Council for Health, Working Life and Welfare; Formas; the Swedish Asthma and Allergy Research Foundation; the Swedish Heart-Lung Foundation; and Region Stockholm (ALF project, and support for cohort and database management). The funding sources had no involvement in study design; collection, analysis, and interpretation of data; writing of the report; or the decision to submit the article for publication.

Disclosure of potential conflict of interest: The authors declare that they have no relevant conflicts of interest.
