# Supplementary material for: Asthma increases short-time sickness absence and presenteeism in young adults
Source: J Allergy Clin Immunol Glob. 2025 Jun 24;4(3):100518. doi: 10.1016/j.jacig.2025.100518 (PMC12281836; doi:10.1016/j.jacig.2025.100518)
Supplement: Supplementary Data [file mmc1.docx]

**Online Repository text**

**Methods**

The study was based on the population-based birth cohort BAMSE, originally including 4,089 children born between 1994 and 1996 in the central and northwestern parts of in Stockholm, Sweden. The study participants have been followed with repeated questionnaires and clinical investigations throughout childhood, adolescence, and up to young adulthood. At around age 24 years (2016-2019), participants answered a questionnaire (n=3,064), including details of asthma and rhinitis. In a follow-up at 28 years (2023), participants answered another questionnaire (n=2,101) including sickness absence and presenteeism.

Short-term sickness absence was defined as episodes with sickness absence <14 days in the last 12 months, categorized as no/once (reference group), 2-4 times and ≥5 times. Presenteeism was defined as working despite being unwell in the preceding 12 months. The response alternatives “a few” and “several times” were combined and compared to the response alternatives “no” and ”one time”, combined (reference group).

At age 24 years, current asthma was defined as self-reported doctor’s diagnosed asthma in combination with breathing difficulties and/or asthma medication use in the preceding 12 months. Allergic rhinitis was defined as eye/nose symptoms from furred animals or pollen without having a cold in the preceding 12 months.

Information on skill level in occupations was collected from the longitudinal integrated database for health insurance and labor market studies (LISA) from the year 2021. Occupation was coded in accordance with Swedish standard Classification of Occupation (SSYK) 2012 and grouped based on skill level into high (skill level 3-4: managerial occupations and occupations with university qualifications [SSYK 1-3]) and low (skill level 1-2: occupations in administration, service, care, and sales work, agriculture, gardening, forestry, fishing, construction, manufacturing, transport, and occupations requiring shorter training (elementary school level) [SSYK 4-9]) ^1^

Analyses were performed with multinominal logistic regression (sickness absence) and logistic regression (presenteeism) adjusted for sex, education (elementary school/upper secondary school, folk high school/university <3 years, or university ≥3 years), employment status (student, worker, or other), smoking (no, occasional, daily) and body mass index. Analyses were further stratified for sex and occupational skill level. Education was excluded in analyses stratified on occupational skill level to avoid overadjustment. Missing data was handled by complete case analysis.

**Reference**

1. <https://www.scb.se/contentassets/0c0089cc085a45d49c1dc83923ad933a/in-english-ssyk-2012.pdf> (accessed April 8th 2025).
